# Diagnosis

**Published:** 1874-02

**Authors:** J. C. Scott


### DIAGNOSIS.

BY J. C. SCOTT, D. D. S.

The importance of exhaustive scrutiny and thoroughness in the examination of teeth previous to filling, was never so palpable to me as when, a few days since, I incidentally made a discovery, after it was too late, that confused me.

It was the first left superior molar that I had in hand. The teeth in front of it had been removed for the purpose of inserting a partial artificial denture. The tooth presented all the external signs of a sound and perfect living molar, in every respect, with the exception of a crown cavity, and an anterior proximal one. With great care I had completed two gold fillings, and felt that I had done myself much credit. But as our most lofty exaltations are sometimes soonest and deepest prostrated, so was it in this case. By the merest incident, I discovered that something was wrong, both on the posterior proximal surface and at the margin of the gum on the palatine surface.

The tooth fit up squarely and accurately against the second molar. The eye could detect no discoloration through the enamel, or breach of continuity, and I made the discovery in attempting to examine the rear tooth. The tooth was decayed in such a manner that the shell easily gave way, revealing the fact that not only a large portion of the dentine was gone, but, to my deep chagrin, and mortification as well, the discovery was made that the tooth was necrosed and had been for some time.

But that which now seems strange and which at the time assured me, was that in burning out the front cavity, the patient, who was a healthy young lady of a little above twenty years, made the same complaint that daily occurs when cutting into sensitive dentine. When using the mallet the patient did not complain in the least, as is generally done in filling a necrosed tooth. Why was this ? Was the dentine of this necrosed tooth sensitive at the cervical margin of the cavity ?

